# Assessment of the use and need for an integrated molecular surveillance of tuberculosis: an online survey in Germany

**DOI:** 10.1186/s12889-019-6631-6

**Published:** 2019-03-18

**Authors:** Andrea Sanchini, Marta Andrés, Lena Fiebig, Stefan Albrecht, Barbara Hauer, Walter Haas

**Affiliations:** 1 Department for Infectious Disease Epidemiology, Robert Koch Institute, Seestraße 15, 13353 Berlin, Germany; 2 Department for Epidemiology and Health Monitoring, Robert Koch Institute, Berlin, Germany; 3 Current address: Ear Institute, University College London, London, UK; 4 Current address: Anti-Persoonsmijnen Ontmijnende Product Ontwikkeling – APOPO, Sokoine University of Agriculture, Morogoro, Tanzania

**Keywords:** Tuberculosis, Molecular surveillance, Epidemiology, Public health, Molecular typing, *Mycobacterium tuberculosis*, Survey, Voxco, Whole genome sequencing

## Abstract

**Background:**

The implementation of an integrated molecular surveillance (IMS) of tuberculosis (TB) is of high priority for TB control. IMS is defined as the systematic inclusion of molecular typing results in the national TB surveillance system. Although not standardized, an IMS of TB is already implemented in several low TB incidence countries. Germany is in the process of implementing a nationwide IMS of TB. This requires close collaboration between national and local health authorities. We conducted an online survey to understand the current use of molecular typing results for TB surveillance among the local public health offices (PHO)s in Germany, and to collect their perception and expectations towards the implementation of a nationwide IMS of TB.

**Methods:**

The online survey was developed using the software Voxco and included 31 questions. The survey was sent to all the 377 local PHOs in Germany in April 2017. Responses were collected until June 2017.

**Results:**

A total of 174/377 (46.2%) local PHOs participated in our survey, and 88/377 (23.3%) used molecular typing results in their routine TB surveillance work. The PHOs used molecular typing results especially as support for epidemiological contact tracing (62/88, 70.4%). We found statistically significant differences between answers of PHOs that *did not use* molecular typing results (*n* = 86) vs. PHOs that *did use* molecular typing results (*n* = 88): the latter perceived the use of molecular typing results as more beneficial for their work compared to the former (65.9% vs. 34.9%, *p* < 0.05). Moreover, the PHOs using molecular typing results expect for the future more support and coordination from regional and national public health institutes, especially regarding the identification and analysis of molecular clusters.

**Conclusions:**

Our study is a step forward in the broader goal of implementing an IMS of TB in Germany. The local PHOs currently using the molecular typing results highlighted their positive attitude towards the implementation of an IMS, but also their needs of more support. Similar assessments might serve as an example for other countries which are on the way to implement a nationwide IMS of TB.

**Electronic supplementary material:**

The online version of this article (10.1186/s12889-019-6631-6) contains supplementary material, which is available to authorized users.

## Background

According to public health institutes in Europe, the implementation of a nationwide integrated molecular surveillance (IMS) is of high priority to improve tuberculosis (TB) control [1]. The IMS is defined as the systematic and automatic inclusion of molecular typing results in the national TB surveillance system. In other words, it means combining the molecular typing results of the *Mycobacterium tuberculosis* (MTB) isolate (such as genetic lineage or drug-resistance determinants) with epidemiological data of TB patients (such as demographics or contacts) [2, 3]. The combination of molecular typing results–especially by using whole genome sequencing (WGS)–with epidemiological data, demonstrated several benefits for TB surveillance and control, such as inferring or excluding transmission during outbreaks [4–6], distinguishing between relapses/reinfection [7, 8], distinguishing between primary/acquired drug resistance [9], evaluating TB control strategies [10] and understanding TB epidemiology [11].

A nationwide IMS of TB, although not standardized, is implemented in few countries such as Denmark [12], England [13], Finland [14], the Netherlands [15], Slovenia [16], Sweden [17] and the United States [18]. Germany is in the process of implementing IMS of TB. In the past years, several studies have been done in Germany in the context of molecular surveillance of TB. However, all these studies have been conducted at the local level (for example in one or two German federal states), were restricted in time and used different molecular typing methods. For example, in a study conducted in 2003–2005 in the federal state of Baden-Württemberg, molecular typing results (obtained using IS6110 DNA fingerprinting and spoligotyping) and the conventional contact tracing information were used to estimate TB transmission in Germany between people with and without a migration background. The results showed that there was no transmission of TB between immigrants originally from high TB prevalence countries and the native population with low TB prevalence [19]. Andrés and co-authors conducted a pilot project in 2008–2010 in the federal state of Baden-Württemberg on the systematic integration of molecular typing results (coming from the National Reference Center for Mycobacteria) in the national TB surveillance system. All 923 culture-positive isolates from Baden-Württemberg were typed using the IS6110 DNA fingerprinting. In 11% of the isolates belonging to a molecular cluster, the molecular typing results identified a link in absence of any epidemiological link. The results of this pilot demonstrated the feasibility and the usefulness of an IMS of TB at a local level [20]. In another study conducted in 2006–2010 in the federal state of Schleswig-Holstein, the authors compared the discriminatory power of three different molecular typing methods for TB, such as IS6110 DNA fingerprinting, spoligotyping and 24-loci mycobacterial interspersed repetitive units (MIRU). The study revealed that in 93.1% of the cases there was concordance among the results and the clustering among these methods [21]. In a longitudinal molecular epidemiology study conducted from 1997 to 2010 in the federal state of Schleswig-Holstein and in the city of Hamburg, researchers compared WGS and other typing methods in analysing an outbreak (86 isolates). The WGS identified seven clusters and 36 unique isolates and demonstrated that five isolates were wrongly included in a cluster by the other typing methods [5]. Fiebig and co-authors investigated in 2014 an international cluster of multidrug-resistant TB spread in Austria, Romania and Germany. Ten isolates were included in one cluster according to the MIRU typing method, however, the WGS further divided these 10 isolates into 2 subgroups, suggesting the presence of two distinct transmission events [22].

Such local studies conducted in Germany can be seen as “building blocks” for the preparation of a nationwide IMS of TB. The implementation of a nationwide IMS of TB is a public health intervention which requires a close collaboration between several players, such as the Robert Koch Institute (RKI, the German national public health institute) acting as coordinator, the regional and local public health offices (PHO)s which collect and transmit the epidemiological data, the laboratories performing the molecular typing and the clinical services which communicate with both health authorities and laboratories.

In this study, we focussed on the perspective of the local PHOs in Germany. We conducted an online survey among the local PHOs in Germany to understand their current use of molecular typing results in their routine TB surveillance work, and also their perception and expectations regarding the implementation of a nationwide IMS of TB. At the time of this study, the use of the molecular typing result was not routinely used and could not be reported. This situation has changed with the amendment of the German infection protection law (paragraphs 9 and 11 of the *Infektionsschutzgesetz, IfSG*) on July 2017; available results of molecular typing now need to be included in the notification. In addition, we wanted to see if there was any difference in perception and expectations on the implementation of the IMS of TB between the PHOs belonging to the federal state of Baden-Württemberg–which participated in the pilot study on the implementation of the IMS of TB [20] – and all the other PHOs. Lastly, we investigated if perception and expectations changed over time, by comparing PHOs using molecular typing results recently (up to 2016) and in the previous years.

## Methods

### Survey development and distribution

We developed an online survey using the software Voxco (Acuity 4 Survey, Voxco, Montreal, Canada). The questionnaire was divided into four parts: 1) general information on each PHO; 2) information regarding the use of the molecular typing result up to 2016; 3) information on the performance of contact tracing and 4) expectations for the implementation of a nationwide IMS of TB. We developed a total of 31 questions. In 19 questions only one answer was possible, in 11 questions multiple answers were possible and one question was an open question. In 10 questions we collected quantitative information such as the number of TB cases per PHO (for details about the online survey see the Additional file 1). The link to the online survey was sent to all 377 PHOs in Germany on the 24th of April 2017. The responses were collected at the RKI until the 16th of June 2017. We asked the PHOs to answer the questions referring to their work up to the 31.12.2016. The Data Protection Officer (*Datenschutzbeauftragte/r, DSB*) and the Information Security Officer (*Informationssicherheitsbeauftragte/r, ISB*) of the RKI examined our study design and gave us their ethical approval, basis on the fact that there were no data protection concerns against the implementation of our study. As our study did not involve interventions no further ethical clearance was necessary.

### Descriptive and analytical approach

We analyzed the response rate country-wide and stratified by German federal state. In addition, we calculated the molecular typing coverage for each federal state, defined by the number of PHOs which used molecular typing results divided by the number of invited PHOs per federal state. We conducted a descriptive analysis of the answers to the survey. As an analytical approach, we compared the qualitative answers to the survey between different groups, specifically between:

1. PHOs that *did not use* molecular typing results vs. PHOs that *did use* molecular typing results;
2. PHOs in the federal state of Baden-Württemberg vs. PHOs in all other federal states;
3. PHOs that used molecular typing results *in 2016* vs. PHOs that used molecular typing results *prior to 2016*.

Pearson’s chi-squared and Fisher’s exact tests were used to determine significant differences in the answers to the survey between the different groups. Statistical significance was considered with a two-sided test if *p* < 0.05. Data were collected using Microsoft Excel and analyzed using STATA version 14.0 (Stata Corp. 2011. Stata Statistical Software: Release 12. College Station, TX: Stata Corp LP).

## Results

### Survey response rate, number of TB cases and contact tracings per PHO

A total of 174/377 (46.2%) of PHOs participated in our online survey. All 16 German federal states were represented with at least one PHO (Table 1). The molecular typing coverage varied substantially among the PHOs belonging to different federal states, ranging from 3.8% in Rhineland-Palatinate to 54.0% in Baden-Württemberg and 43.7% in Schleswig-Holstein (Table 1). The median number of notified TB cases per participating PHO in 2016 was 13.5 (ranging from 0 to 396). The median number of contact tracings, meaning the number of individual contacts traced, per PHO in 2016 was 116 (ranging from 2 to 2173). Therefore, for each TB case, a median of 8.6 contacts were traced.

**Table 1:** Survey participation and molecular typing rates of local PHO by German federal state

Federal State	Participating local PHOs (n)	Invited local PHOs (N)	Participation rate, % (n/N)	Local PHOs using molecular typing results (n1)	Molecular typing coverage, % (n1/N)
North Rhine-Westphalia	33	53	62.3	15	24.1
Baden-Württemberg	23	37	62.2	20	54.0
Bavaria	22	76	28.9	14	18.4
Lower Saxony	16	44	36.3	7	15.9
Hesse	13	25	52.0	4	16.0
Schleswig-Holstein	12	16	75.0	7	43.7
Brandenburg	11	21	52.4	4	19.0
Thuringia	9	24	37.5	5	20.8
Saxony-Anhalt	8	12	66.7	2	16.7
Unknown^a^	7	7	100.0	1	14.3
Mecklenburg-Vorpommern	6	8	75.0	2	25.0
Saxony	5	13	38.5	3	23.1
Saarland	5	6	83.4	1	16.7
Berlin	1	1	100.0	0	0.0
Bremen	1	1	100.0	1	100.0
Hamburg	1	7	14.3	1	14.3
Rhineland-Palatinate	1	26	3.8	1	3.8
Total	174	377	46.2	88	23.3

*PHO* Public health office

^a^ In seven cases, we had no information about the geographical location of the local PHO

### Descriptive analysis of the survey answers among all PHOs

A total of 88/377 (23.3%) PHOs used the molecular typing results in their routine TB surveillance work in 2016 or prior to 2016. The molecular typing was performed in 76/88 (85.2%) cases at the German National Reference Centre for Mycobacteria (Forschungszentrum Borstel, Borstel, Germany), in 6/88 (6.8%) cases at other laboratories, in four cases at multiple places and in two cases this information was unknown. The most common molecular typing method analyzed was spoligotyping in 41/88 (46.6%), followed by MIRU in 26/88 (29.5%), WGS in 7/88 (7.9%) and multiple methods in 14/88 (15.9%). The costs for the molecular typing were covered by the local PHO in two-thirds of cases (59/88, 67.0%) or by the laboratory performing the typing (11/88, 12.5%). The estimated median cost for the PHO per typed isolate was 50 Euro (ranging from 0 to 360 Euro), considering all the typing methods. The median turnaround time per isolate, defined as the time taken from the request to the delivering of the molecular typing result, was 21 days (ranging from 7 to 90 days, IQR1 = 14, IQR3 = 28). Among the PHOs that used molecular typing results, the majority (49/88, 55.7%) did not systematically document such results in their TB-surveillance reporting system.

We asked the PHOs: “For which purpose did you use the molecular typing result?” The most common answer was “support for epidemiological contact tracing” 62/88 (70.4%), highlighting the added value of combining molecular typing and epidemiological data in the control of TB transmission. Among the other purposes, the PHOs reported: distinguish between reinfection-relapses (18/88, 20.4%), molecular detection of drug-resistance (10/88, 11.4%) or detection of false-positive laboratory results (4/88, 4.5%). We further elaborated on this question and asked: “Which were the benefits of using the molecular typing results for your work?” The answers of the PHOs reflected once again the advantages of combining molecular typing and epidemiological data: detection of unknown transmission (41/88, 46.6%), transmission detection over longer time period (37/88, 42.0%), exclusion of previously assumed transmission (33/88, 37.5%) early detection of outbreaks (25/88, 28.4%) or detection of transregional transmission (24/88, 27.3%). Among the PHOs participating in our survey, the major barriers to the implementation of the IMS of TB were: costs (123/174, 70.7%), lack of staff capacity (57/174, 32.8%) and complex logistics related to sample shipping (45/174, 25.9%).

A total of 38 PHOs answered that they did not perceive the IMS of TB as beneficial (or only partially beneficial, see question 4.1 in the Additional file 2). The specific reasons for such answers were: “rarity of TB cases”, “the molecular typing result was not required” (see below), or “no effect of the molecular typing results in the practical work”, since the pathway of transmission of infection was already known due to epidemiological information.

### Comparisons of answers among the different groups of PHOs

**Fig. 1:**
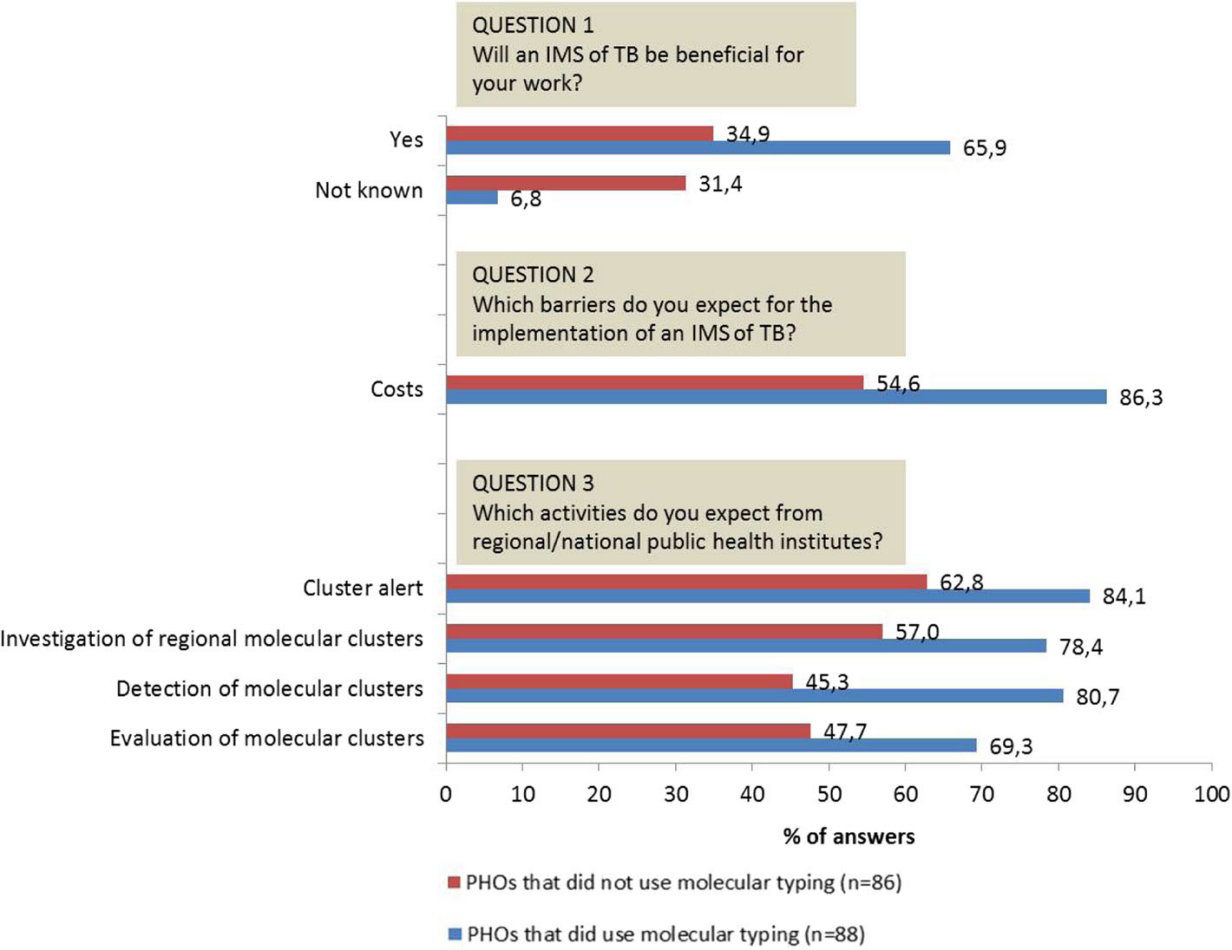
Answers of local public health offices that *did not use* vs. *did use* molecular results. In this figure, only the differences in answers which were statistically significant between the two groups (*p* < 0.05) are showed. For an overview of all the possible answers, please refer to the Additional files 1 and 2. PHO: Public health office; IMS: integrated molecular surveillance; TB: Tuberculosis

In the Additional file 2, the answers to the survey between different groups of PHOs are reported. We found statistically significant differences by comparing local PHOs that *did not use* molecular typing results (*n* = 86) vs. local PHOs that *did use* molecular typing results (*n* = 88). Indeed, the group that used molecular typing results perceived it more often as beneficial for their work compared to the other group (Fig. 1, question 1). Moreover, the group that did not use the molecular typing results answered more often that they do not know if a nationwide IMS of TB will be beneficial for their routine work (Fig. 1, question 1). The PHOs using molecular typing results declared also more often that cost is the major barrier to the implementation of an IMS of TB (Fig. 1, question 2). The strongest differences between these two groups were that PHOs using molecular typing results expected more support and coordination from regional and national public health institutes such as the RKI – especially regarding the identification of molecular clusters (Fig. 1, question 3).

We analyzed the answers of the PHOs in the federal state of Baden-Württemberg since in this federal state a pilot study on the feasibility of the IMS was carried out and we wanted to see their perception about the IMS [20]. The PHOs in the federal state of Baden-Württemberg responded more positively to the question “will an IMS of TB be beneficial for your work?” when compared with all other PHOs (18/23, 78.3% vs. 70/151, 46.4%, *p* < 0.05). Another difference between these two groups is that PHOs in Baden-Württemberg expect more support in the identification of molecular cluster when compared to all other PHOs (23/23, 100% vs. 87/151, 57.6%, *p* < 0.05).

**Fig. 2:**
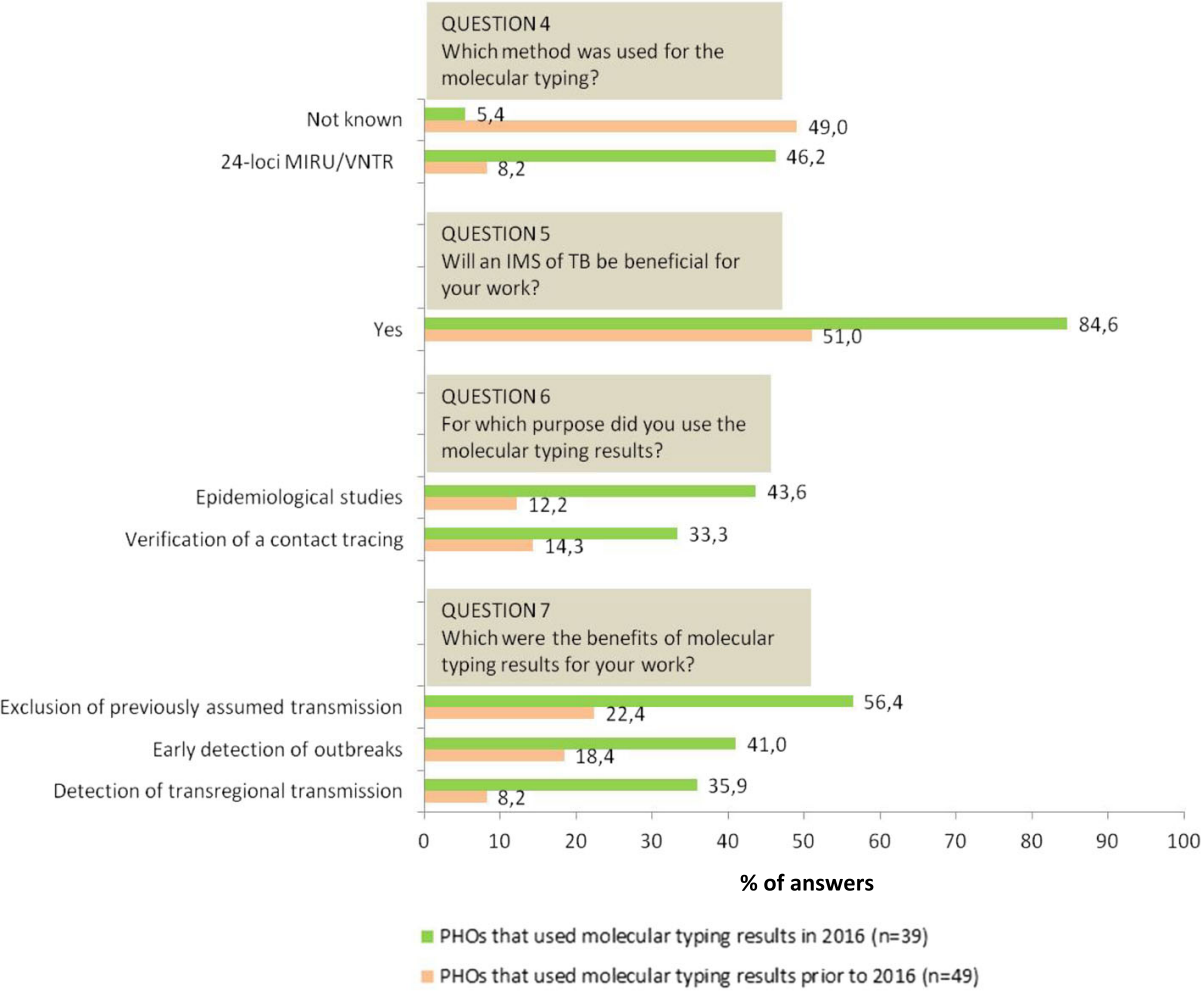
Answers of local public health offices that used molecular results *in 2016* vs. *prior to 2016*. In this figure, only the differences in answers which were statistically significant between the two groups (*p* < 0.05) are showed. For an overview of all the possible answers, please refer to the Additional files 1 and 2. PHO: Public health office; IMS: integrated molecular surveillance; TB: Tuberculosis

Among the PHOs that did use molecular typing results, we investigated also if there were any changes in the answers over the time in perception and in expectations. The PHOs that used molecular typing results *in 2016* (*n* = 39) perceived it as more beneficial for their work compared to the PHOs that used molecular typing results prior *to 2016* (*n* = 49) (Fig. 2, question 5). The PHOs that used the molecular results *in 2016* also used these results for more purposes, such as epidemiological studies or to verify retrospectively a contact of a TB index patient (Fig. 2, question 6). In addition, they perceived also more benefits for their own work, such as “exclusion of previously assumed transmission”, “early detection of outbreaks” and “detection of transregional transmission” (Fig. 2, question 7). We found a difference also regarding the molecular typing methods analyzed: the PHOs that used molecular typing results *in 2016* analyzed more often the results of the MIRU molecular typing method in comparison with the PHOs that used molecular typing results prior *to 2016* (Fig. 2, question 4).

## Discussion

In this study, we conducted a survey among the German local PHOs to assess their use and needs of molecular typing results for TB surveillance, and their expectations of an IMS of TB in Germany. These objectives fit within the broader goal of implementing a nationwide IMS of TB in Germany.

We observed a high variability in the use of molecular typing results for TB surveillance among the PHOs in Germany. We expected such variability since the IMS is not yet implemented nationally. Around a quarter of the PHOs invited to our survey used the molecular typing results in their routine work. The PHOs belonging to the federal states of Baden-Württemberg and Schleswig-Holstein have high molecular typing coverages and also high participation rates to our survey. These high molecular typing coverages and participation rates might be explained by the fact that the PHOs in those two federal states already participated in studies combining molecular typing results and epidemiological data [5, 19–21]. The stratified analysis on the PHOs belonging to Baden-Württemberg highlighted a strong positive perception on the implementation of the IMS of TB but also a need of more support in the identification of molecular clusters. This outcome might be the consequence of the training and of the support provided by the RKI and the other collaborators during the pilot study on the feasibility of the IMS carried out in Baden-Württemberg between 2008 and 2010 [21]. This positive outcome of the pilot might have implications for a successful implementation of the IMS of TB in Germany.

In our study, we also observed a high variability of the molecular typing methods used for MTB. The low proportion of WGS observed might be explained by the fact that some PHOs answering our survey used molecular typing results also prior to 2016, and therefore they might not be already updated to the WGS. In addition, other typing methods might still be cheaper and more standardized (such as the MIRU) compared to the WGS. This is true also for the other countries where the IMS of TB was implemented in the previous years: for example in the Netherlands, Slovenia, Sweden and the United States, the molecular typing method used was the MIRU [15–18], while in Finland and Denmark the method used was spoligotyping or IS6110 DNA fingerprint [12, 14]. To our knowledge, England is the only country where, from March 2017, a routine WGS service has been implemented for all MTB isolates [23]. However, a recent survey conducted by the RKI among the TB National Focal Points in Europe revealed that many other countries such as Austria, Denmark, Finland and Norway seem to be in the transition phase towards the implementation of a routine WGS service (data not shown).

Among the participating PHOs using molecular typing results, less than half included such results systematically in their TB surveillance system, which is a prerequisite to implementing a nationwide IMS of TB. This challenge can be solved by providing training about the benefits of the IMS and of the systematic inclusion of the molecular typing results in the TB surveillance system.

We observed that a third of PHOs that did not use molecular typing results answered that they did not know if a nationwide implementation of the IMS of TB would be beneficial for their work. In addition, several PHOs answered that they did not perceive the IMS of TB as beneficial for their routine work. These answers might reflect the need for training and education on the benefits of the IMS of TB. The PHOs that used molecular tying results were indeed more aware of the benefits of IMS of TB compared to the PHOs that did not use molecular typing results. We observed a similar trend between the PHOs that used it in 2016, compared to those using it prior to 2016. Therefore there seems to be a growing perception of the benefits of the IMS over time, and this might be due to several factors: the growing experience in the interpretation of molecular typing results, the availability of more advanced and faster methodologies and the increased education and awareness about the IMS of TB. The major benefits identified by the responding PHOs in our study were the support in transmission detection, contact tracing and cluster investigations in outbreaks. Similar results were obtained also in a study in the Netherlands assessing the impact of molecular typing on contact tracing [24]. Two studies in England highlighted also a positive perception regarding the implementation of an IMS of TB [25, 26]. Indeed, the benefits described in these two studies are similar to those identified in our study: understanding TB epidemiology, confirming/refuting suspected transmission or supporting outbreak investigation. Such benefits can have an impact on public health and save public health money, for example by avoiding unnecessary contacts tracing or by targeting preventive measures [23, 27]. PHOs using molecular typing results were also more aware of the barriers, particularly costs, to the implementation of a nationwide IMS of TB. In addition, they clearly highlighted their need for more support and coordination from regional or national public health institutes, in data interpretation, especially regarding the support for molecular cluster identification. In our opinion, this is a clear need identified from the local PHOs which have direct practical experience with the molecular surveillance of TB and it will be one of our main tasks to fulfil this need as national public health institute.

One of the limitations of our study is that the PHOs who used molecular typing results might have been more willing to participate in our survey and to provide answers, therefore leading to an overestimation of the use of the molecular typing results. Moreover, prior experience may have also influenced responses due to greater insight and education on the value of the IMS in practice. In addition, PHOs using molecular typing results in 2016 might also remember more answers and details compared to the ones that used them prior to 2016. Moreover, we did not ask the PHOs if they ever received the molecular typing results but they did not use them. This could have added further insights on the usefulness of the molecular typing results for the PHOs. Lastly, around half of the invited PHOs did not participate in our study, highlighting the need for more training and awareness on the importance of a national IMS of TB. Possible reasons to explain why certain PHOs did not participate in our survey might be the rarity of TB cases in certain PHOs and also the fact that the molecular typing result was not part of the official notification, thus making the PHO less motivated to participate in such type of survey.

## Conclusions

Our study is a step forward in the broader goal of implementing a nationwide IMS of TB in Germany. The answers of the local PHOs to our survey highlighted their positive attitude towards the implementation of an IMS, but also their needs of more support in data interpretation. Therefore, a closer collaboration is needed between local and national public health authorities. For example, molecular typing data from different PHOs should be made more accessible, organized and centralized, or regular training workshops on molecular surveillance could be organized. Such closer collaboration might also encourage and convince those PHOs in Germany, who do currently not use molecular typing results in their work, of the benefits of the IMS. In Germany, the concept of implementing a nationwide IMS of TB started years ago with smaller and local molecular surveillance studies–the so-called building blocks in our introduction–which helped identify specific needs and room for improvements first at a smaller scale. After these studies, we are now in a good position to launch a nationwide IMS of TB. Such an approach could be used also by other countries, which are on the way to develop a nationwide IMS of TB.

## Additional files

Additional file 1: Word format of the online survey. (DOCX 43 kb)

Additional file 2: Qualitative answers to the survey between different groups of PHOs. (XLSX 17 kb)

## Abbreviations

IMS: Integrated molecular surveillance
MIRU: Mycobacterial interspersed repetitive units
MTB: *Mycobacterium tuberculosis*
PHO: Public health office
RKI: Robert Koch Institute
TB: Tuberculosis
WGS: Whole genome sequencing

## References

[1] ECDC. ECDC roadmap for integration of molecular and genomic typing into European-level surveillance and epidemic preparedness ECDC TECHNICAL REPORT. 2016. https://ecdc.europa.eu/sites/portal/files/media/en/publications/Publications/molecular-typing-EU-surveillance-epidemic-preparedness-2016-19-roadmap.pdf. Accessed 8 Dec 2017.

[2] Hatherell HA, Colijn C, Stagg HR, Jackson C, Winter JR, Abubakar I (2016). Interpreting whole genome sequencing for investigating tuberculosis transmission: a systematic review. BMC Med.

[3] Nikolayevskyy V, Kranzer K, Niemann S, Drobniewski F (2016). Whole genome sequencing of *Mycobacterium tuberculosis* for detection of recent transmission and tracing outbreaks: a systematic review. Tuberculosis..

[4] Gardy JL, Johnston JC, Ho Sui SJ, Cook VJ, Shah L, Brodkin E (2011). Whole-genome sequencing and social-network analysis of a tuberculosis outbreak. New Engl J Med.

[5] Roetzer A, Diel R, Kohl TA, Ruckert C, Nubel U, Blom J (2013). Whole genome sequencing versus traditional genotyping for investigation of a *Mycobacterium tuberculosis* outbreak: a longitudinal molecular epidemiological study. PLoS Med.

[6] Bryant JM, Schurch AC, van Deutekom H, Harris SR, de Beer JL, de Jager V (2013). Inferring patient to patient transmission of *Mycobacterium tuberculosis* from whole genome sequencing data. BMC Infect Dis.

[7] Bryant JM, Harris SR, Parkhill J, Dawson R, Diacon AH, van Helden P (2013). Whole-genome sequencing to establish relapse or re-infection with *Mycobacterium tuberculosis*: a retrospective observational study. Lancet Resp Med.

[8] Guerra-Assuncao JA, Houben RM, Crampin AC, Mzembe T, Mallard K, Coll F (2015). Recurrence due to relapse or reinfection with *Mycobacterium tuberculosis*: a whole-genome sequencing approach in a large, population-based cohort with a high HIV infection prevalence and active follow-up. J Infect Dis.

[9] Casali N, Nikolayevskyy V, Balabanova Y, Harris SR, Ignatyeva O, Kontsevaya I (2014). Evolution and transmission of drug-resistant tuberculosis in a Russian population. Nat Genet.

[10] de Vries G, van Hest RA, Richardus JH (2007). Impact of mobile radiographic screening on tuberculosis among drug users and homeless persons. Am J Resp Crit Care medicine.

[11] Borgdorff MW, van Soolingen D (2013). The re-emergence of tuberculosis: what have we learnt from molecular epidemiology?. Clin Microbiol Infect.

[12] Kamper-Jorgensen Z, Andersen AB, Kok-Jensen A, Bygbjerg IC, Andersen PH, Thomsen VO (2012). Clustered tuberculosis in a low-burden country: nationwide genotyping through 15 years. J Clin Microbiol.

[13] Lalor MK, Anderson LF, Hamblion EL, Burkitt A, Davidson JA, Maguire H (2017). Recent household transmission of tuberculosis in England, 2010-2012: retrospective national cohort study combining epidemiological and molecular strain typing data. BMC Med.

[14] Raisanen PE, Soini H, Vasankari T, Smit PW, Nuorti JP, Ollgren J (2016). Tuberculosis in immigrants in Finland, 1995-2013. Epidemiol Infect.

[15] Sloot R, Borgdorff MW, de Beer JL, van Ingen J, Supply P, van Soolingen D (2013). Clustering of tuberculosis cases based on variable-number tandem-repeat typing in relation to the population structure of *Mycobacterium tuberculosis* in the Netherlands. J Clin Microbiol.

[16] Bidovec-Stojkovic U, Zolnir-Dovc M, Supply P (2011). One year nationwide evaluation of 24-locus MIRU-VNTR genotyping on Slovenian *Mycobacterium tuberculosis* isolates. Resp Med.

[17] Jonsson J, Hoffner S, Berggren I, Bruchfeld J, Ghebremichael S, Pennhag A (2014). Comparison between RFLP and MIRU-VNTR genotyping of *Mycobacterium tuberculosis* strains isolated in Stockholm 2009 to 2011. PLoS One.

[18] Shea KM, Kammerer JS, Winston CA, Navin TR, Horsburgh CR (2014). Estimated rate of reactivation of latent tuberculosis infection in the United States, overall and by population subgroup. Am J Epidemiol.

[19] Barniol J, Niemann S, Louis VR, Brodhun B, Dreweck C, Richter E (2009). Transmission dynamics of pulmonary tuberculosis between autochthonous and immigrant sub-populations. BMC Infect Dis.

[20] Andres M, Gohring-Zwacka E, Fiebig L, Priwitzer M, Richter E, Rusch-Gerdes S (2017). Integration of molecular typing results into tuberculosis surveillance in Germany-a pilot study. PLoS One.

[21] Roetzer A, Schuback S, Diel R, Gasau F, Ubben T, di Nauta A (2011). Evaluation of *Mycobacterium tuberculosis* typing methods in a 4-year study in Schleswig-Holstein, Northern Germany. J Clin Microbiol.

[22] Fiebig L, Kohl TA, Popovici O, Mühlenfeld M, Indra A, Homorodean D, et al. A joint cross-border investigation of a cluster of multidrug-resistant tuberculosis in Austria, Romania and Germany using classic, genotyping and whole-genome sequencing methods: lessons learnt. Euro Surveill. 2017;22 pii=30439. 10.2807/1560-7917.ES.2017.22.2.30439 PMC5404487 28106529

[23] Walker TM, Cruz AL, Peto TE, Smith EG, Esmail H, Crook DW (2017). Tuberculosis is changing. Lancet Infect Dis.

[24] Lambregts-van Weezenbeek CS, Sebek MM, van Gerven PJ, de Vries G, Verver S, Kalisvaart NA (2003). Tuberculosis contact investigation and DNA fingerprint surveillance in the Netherlands: 6 years' experience with nation-wide cluster feedback and cluster monitoring. Int J Tuberc Lung D.

[25] Mears J, Abubakar I, Crisp D, Maguire H, Innes JA, Lilley M (2014). Prospective evaluation of a complex public health intervention: lessons from an initial and follow-up cross-sectional survey of the tuberculosis strain typing service in England. BMC Public Health.

[26] Mears J, Vynnycky E, Lord J, Borgdorff MW, Cohen T, Crisp D (2016). The prospective evaluation of the TB strain typing service in England: a mixed methods study. Thorax..

[27] Walker TM, Ip CL, Harrell RH, Evans JT, Kapatai G, Dedicoat MJ (2013). Whole-genome sequencing to delineate *Mycobacterium tuberculosis* outbreaks: a retrospective observational study. Lancet Infect Dis.

