# Supplementary material for: Assessment of the use and need for an integrated molecular surveillance of tuberculosis: an online survey in Germany
Source: BMC Public Health. 2019 Mar 18;19:321. doi: 10.1186/s12889-019-6631-6 (PMC6423790; doi:10.1186/s12889-019-6631-6)
Supplement: Supplementary file 1 — Word format of the online survey. (DOCX 43 kb) [file 12889_2019_6631_MOESM1_ESM.docx]

**Part 1: General information on each local public health office (PHO)**

**1.1 For how many local PHOs are you responsible for as a Tuberculosis (TB) contact person?**

- - - 1
    - 2
    - 3
    - 4
    - 5

**1.2 Names of these local PHOs**

- - - 1_____________
    - 2_____________
    - 3_____________
    - 4_____________
    - 5_____________

**1.3 How many cases of TB have been reported in your PHO in 2016?**

- - - _____________
    - None
    - Not known
    - Not applicable

**Part 2: Information regarding the use of the molecular typing result**

This part of the questionnaire deals with the current use of molecular typing results for TB surveillance in your local PHO.

**2.1 Did you ever use molecular typing results of *Mycobacterium tuberculosis* complex in your PHO?**

- - - Yes → go to question 2.2, part A
    - No → go to question 2.13, part B
    - Not known → go to question 2.13, part B
    - Not applicable → go to question 2.13, part B

**Part A: Questions to local PHOs that *did use* molecular typing results**

**2.2 Did it ever happen that you needed the molecular typing results, but they were not available?**

- - - Yes → go to question 2.2.1
    - No → go to question 2.3
    - Not known → go to question 2.3
    - Not applicable → go to question 2.3

**2.2.1 For which reason?**

(*Multiple answers possible*)

- - - The molecular typing was required, but no result was available
    - The molecular typing was not possible due to (*please specify*): ______________________________
    - Others (*please specify*): ______________________________
    - Not known
    - Not applicable

**2.3 For how many cases of TB reported in 2016 did you have a molecular typing result?**

- - - **____________**
- None
  - - Not known
    - Not applicable

**2.3.1 In which year (until 31.12.2016) did you use the molecular typing results of *Mycobacterium* *tuberculosis* complex?**

- _____________Year
- Not known
- Not applicable

**2.4 Where was the molecular typing performed?**

(*Multiple answers possible*)

- - - National Reference Center for Mycobacteria
- Other laboratories, (*please specify*) _______________________________________________
  - - Not known
    - Not applicable

**2.5 Which method was used for the molecular typing?**

(*Multiple answers possible*):

- - - IS6110-RFLP
    - Spoligotyping
    - 24-loci MIRU/VNTR
    - Whole genome sequencing
    - Others *(please specify):* ____________________________
    - Not known
    - Not applicable

**2.6 Who took the costs of molecular typing?**

(*Multiple answers possible*)

- The local PHO
- The laboratory
- The accident or working health insurance
- The medical institution responsible for the patients
- Others *(please specify):* ____________________________
- Not known
- Not applicable

**2.7 What was the average cost per typed isolate?**

- - - **____________**Euro pro typed isolate
    - Not known
    - Not applicable

**2.8 How long was the average time span from the request to the delivery of the molecular typing results?**

- - - **____________**Days
    - Not known
    - Not applicable

**2.9 How long would the desirable time span be from the request to the delivery of the molecular typing results?**

- - - **________________**Days
    - Not known
    - Not applicable

**2.10 Were the molecular typing results systematically documented in your TB surveillance reporting software?**

- - - Yes → go to question 2.10.1
    - No → go to question 2.11
    - Not known → go to question 2.11
    - Not applicable → go to question 2.11

**2.10.1 How were the molecular typing results documented in your TB surveillance reporting software?**

- As a complete typing result
- In the form of a result indicator
- In the form of a mark for a molecular cluster
- Others (*please specify*):______________________________________________________
  - - Not known
    - Not applicable

**2.11 For which purpose did you use the molecular typing results?**

*(Multiple answers possible)*

- Supporting the conduct of a contact tracing
- Verification of a contact tracing (retrospective)
- Recognizing false positive culture outcomes
- Individual medical benefit, such as differentiation between reinfection and reactivation
- Epidemiological studies
- Genotypic resistance determination
- Others (*please specify*):____________________________________________________________
  - - Not known
    - Not applicable

**2.12 Which were the benefits of molecular typing results for your work?**

*(Multiple answers possible)*

- - - Exclusion of previously assumed transmission
    - Early detection of outbreaks
    - Detection of trans-regional transmission
    - Transmission Detection over a longer period
    - Recognizing a false positive culture result
    - Genotypic resistance determination
- Epidemiological knowledge
- Others (*please specify*):____________________________________________________________
- No benefit
- Not known
  - - Not applicable

**Part B: Questions to local PHOs that *did not* use molecular typing results**

**2.13 Did it ever happen that you needed the molecular typing results, but they were not available?**

- - - Yes → go to question 2.13.1
    - No → go to question 2.14
    - Not known → go to question 2.14
    - Not applicable → go to question 2.14

**2.13.1 For which reason?**

*(Multiple answers possible)*

- - - The request was made, but no result was available
    - The request was not possible due to *(please specify):* ______________________________
    - Others *(please specify):* ______________________________
    - Not known
    - Not applicable

**2.14 For which reasons have the molecular typing results of *Mycobacterium* *tuberculosis* complex not been used in your PHO?**

*(Multiple answers possible)*

- - - Limited financial resources
    - Complex logistics
    - Costs of sample shipment
    - Limited data management resources
    - Lack of staff capacity
    - Lack of capacity to assess additional information
- Others *(please specify)*:____________________________________________________________
  - - No need
    - Not known
    - Not applicable

**3 information on the performance of contact tracing**

This part of the questionnaire contains information regarding the contact tracing for TB cases in your local PHO.

**3.1 How many contact tracings have been performed in your local PHO?**

- - - _______________ → go to question 3.2
    - None → go to question 3.3
    - Not known → go to question 3.3
    - Not applicable → go to question 3.3

**3.2 How many contact tracings were carried out in your PHO in 2016 together with other PHOs?**

- - - **____________** → go to question 3.2.1
    - None → go to question 3.3
    - Not known → go to question 3.3
    - Not applicable → go to question 3.3

**3.2.1 Of the contact tracing carried out in 2016 in collaboration with other PHOs:**

**- How many were carried out in cooperation with other PHOs in the same federal state?**

- - - **____________**
    - None
    - Not known
    - Not applicable

**- How many were carried out in cooperation with other PHOs in another federal state?**

- - - **____________**
    - None
    - Not known
    - Not applicable

**-** **How many were carried out in collaboration with multiple PHOs?**

- - - **____________**
    - None
    - Not known
    - Not applicable

**3.3 How did you document the results of contact tracing?**

*(Multiple answers possible)*

- - - Electronically, within the reporting software
    - Electronically, within another software; Name of the software (*please specify*): __________
    - Paper-based
    - Others (*please specify*):_____________________
    - Not known
    - Not applicable

**4 Expectations for an Integrated Molecular Surveillance of Tuberculosis**

This part of the questionnaire addresses your perceived need for the molecular typing results in the surveillance and control of tuberculosis, and your expectations for a nationwide Integrated Molecular Surveillance (IMS) of Tuberculosis in Germany.

**4.1 Will an IMS of TB be beneficial for your work?**

- - - Yes, as follows (*please specify*): ___________________________
    - No, due (*please specify*):________________________________
    - Partially, as follows (*please specify*): ___________________________
    - Not known
    - Not applicable

**4.2 Which barriers do you expect for the implementation of an IMS of TB?**

*(Multiple answers possible)*

- Costs
- Complex logistics of sample shipment
- Costs of sample shipment
- Limited data management resources
- Lack of staff capacity
- Lack of capacity to assess additional information
- Others (*please specify*)___________________________________
- None
  - - Not known
    - Not applicable

**4.3 Which activities do you expect from regional/national public health institutes?**

*(Multiple answers possible)*

- - - Detection of molecular clusters
      - Informing the responsible local public health office about detected molecular clusters (cluster alert)
      - Evaluation of molecular clusters
    - Investigation of regional molecular cluster
    - Others (*please specify*):______________________________________________________
    - No expectations
    - Not known
    - Not applicable

**4.4 Which further suggestions do you have for the design of an IMS of TB in Germany?**
